# Promoting Collaborative Scholarship During the COVID-19 Pandemic Through an Innovative COVID-19 Data Explorer and Repository at Yale School of Medicine: Development and Usability Study

**DOI:** 10.2196/52120

**Published:** 2024-09-03

**Authors:** Angela Maria Victoria-Castro, Tanima Arora, Michael Simonov, Aditya Biswas, Jameel Alausa, Labeebah Subair, Brett Gerber, Andrew Nguyen, Allen Hsiao, Richard Hintz, Yu Yamamoto, Robert Soufer, Gary Desir, Francis Perry Wilson, Merceditas Villanueva

**Affiliations:** 1 Clinical and Translational Research Accelerator Yale School of Medicine, Yale University New Haven, CT United States; 2 Department of Pediatrics Yale School of Medicine, Yale University New Haven, CT United States; 3 Yale Center for Clinical Investigation Yale School of Medicine, Yale University New Haven, CT United States; 4 Department of Internal Medicine Yale School of Medicine, Yale University New Haven, CT United States; 5 Section of Nephrology Yale School of Medicine, Yale University New Haven, CT United States; 6 Section of Infectious Diseases Yale School of Medicine, Yale University New Haven, CT United States

**Keywords:** COVID-19, database, data access, interdepartmental communication, collaborative scholarship, clinical data, repository, researchers, large-scale database, innovation

## Abstract

**Background:**

The COVID-19 pandemic sparked a surge of research publications spanning epidemiology, basic science, and clinical science. Thanks to the digital revolution, large data sets are now accessible, which also enables real-time epidemic tracking. However, despite this, academic faculty and their trainees have been struggling to access comprehensive clinical data. To tackle this issue, we have devised a clinical data repository that streamlines research processes and promotes interdisciplinary collaboration.

**Objective:**

This study aimed to present an easily accessible up-to-date database that promotes access to local COVID-19 clinical data, thereby increasing efficiency, streamlining, and democratizing the research enterprise. By providing a robust database, a broad range of researchers (faculty and trainees) and clinicians from different areas of medicine are encouraged to explore and collaborate on novel clinically relevant research questions.

**Methods:**

A research platform, called the Yale Department of Medicine COVID-19 Explorer and Repository (DOM-CovX), was constructed to house cleaned, highly granular, deidentified, and continually updated data from over 18,000 patients hospitalized with COVID-19 from January 2020 to January 2023, across the Yale New Haven Health System. Data across several key domains were extracted including demographics, past medical history, laboratory values during hospitalization, vital signs, medications, imaging, procedures, and outcomes. Given the time-varying nature of several data domains, summary statistics were constructed to limit the computational size of the database and provide a reasonable data file that the broader research community could use for basic statistical analyses. The initiative also included a front-end user interface, the DOM-CovX Explorer, for simple data visualization of aggregate data. The detailed clinical data sets were made available for researchers after a review board process.

**Results:**

As of January 2023, the DOM-CovX Explorer has received 38 requests from different groups of scientists at Yale and the repository has expanded research capability to a diverse group of stakeholders including clinical and research-based faculty and trainees within 15 different surgical and nonsurgical specialties. A dedicated DOM-CovX team guides access and use of the database, which has enhanced interdepartmental collaborations, resulting in the publication of 16 peer-reviewed papers, 2 projects available in preprint servers, and 8 presentations in scientific conferences. Currently, the DOM-CovX Explorer continues to expand and improve its interface. The repository includes up to 3997 variables across 7 different clinical domains, with continued growth in response to researchers’ requests and data availability.

**Conclusions:**

The DOM-CovX Data Explorer and Repository is a user-friendly tool for analyzing data and accessing a consistently updated, standardized, and large-scale database. Its innovative approach fosters collaboration, diversity of scholarly pursuits, and expands medical education. In addition, it can be applied to other diseases beyond COVID-19.

## Introduction

COVID-19, the disease caused by the SARS-CoV-2, emerged as a global pandemic that has challenged a broad range of researchers to rapidly develop a new understanding of epidemiology; basic and clinical science to implement novel approaches for prevention and care. There has been an explosion of articles both in the preprint and peer-reviewed literature as evidenced by over 35,000 PubMed-indexed publications within the first 6 months of the pandemic and more than 90,000 by the end of the first year.

The initial reports on COVID-19 from China, describing clinical presentation, diagnosis, and mortality risk factors, were multiauthor collaborations from hospitals and academic centers [1-4]. Subsequent reports included other research and public health stakeholders. Clinical series in the United States evolved to include large multicenter patient cohorts from multiple academic centers and large health systems [5,6]. This focus on large databases led to the exclusion of many clinically intensive frontline clinicians from the research and scholarly enterprise. Many of these were academic physicians whose roles and career paths required scholarly productivity and training of fellows, residents, and students. During the pandemic, time, energy, and other resources were scarce to do scholarly work, which limited the further evaluation of their research hypotheses. These faculty and their trainees had the unique opportunity to generate research questions based on first-hand observations, but without additional support, some questions went uninvestigated [7,8]. Other primary research faculty created proprietary clinical databases to answer research questions relevant to their field but the lack of cross-examination with frontline clinicians and colleagues in other disciplines compromised the depth of analysis. This affected the institutional climate, already stressed by the pandemic, as the valuable input from diverse faculty and trainees was not harnessed for the advancement of knowledge.

Our own local COVID-19 response plan at Yale University School of Medicine (YSM) started in March 2020 and was organized by leadership at YSM and Yale New Haven Health System (YNHHS). The detailed orchestration of efforts involved close interplay between frontline clinicians (academic faculty and hospital staff members) and colleagues experienced in clinical data analysis. Other clinical faculty and trainees who were making key clinical observations expressed a desire to produce scholarly work but were hampered by the lack of a readily available and comprehensive clinical data repository. Various groups tried to create their own databases with manual data extraction from the electronic health record (EHR), but the clinical workload did not allow for timely data management and led to duplication of data collection efforts.

Feedback to leadership from a diverse group of faculty members in the Department of Medicine (DOM) revealed that this lack of access to clinical data resulted in frontline clinicians’ perceptions of being excluded from the research field. Thus, DOM leadership and a subgroup of faculty members (clinicians, clinical researchers, and informatics specialists) met to discuss different approaches. Ultimately, the group agreed on a DOM-supported innovative approach to creating a real-time, clinically comprehensive, single clinical data repository on patients admitted for COVID-19 dating from the start of the pandemic. A distinguishing feature was the availability of a real-time explorer aside from the data repository itself. Through our interface’s statistical visualizations, we offered the opportunity to evaluate certain clinical variables of interest, while researchers were building their hypotheses and adjusting their research proposals. In addition, the request process for access to the data repository was streamlined and simplified, which removed barriers to research activities. In this paper, we aim to describe the creation and dissemination of the Department of Medicine COVID-19 Explorer and Repository (DOM-CovX) which was envisioned to serve as a starting point for clinical queries that could lead to projects ranging from quality improvement to scholarly efforts. We speculate on how this innovation serves to “democratize” the research enterprise by engaging diverse researchers and trainees and promoting a broader scope of scholarship activities.

## Methods

### Construction of the DOM-CovX

YNHHS encompasses 5 hospitals located across Connecticut and Rhode Island with a combined capacity for 2681 inpatient beds. The participating hospitals work together with YSM which oversees both clinical and nonclinical faculty. All sites use Epic as the joint EHR for documentation of clinical care issues.

The Clinical and Translational Research Accelerator (CTRA) team at Yale DOM, comprised of physicians and scientists committed to patient-oriented research and developing solutions in clinical medicine, designed the foundation for the database that led to the development of DOM-CovX. CTRA was initially developed in the Section of Nephrology and early data collection focused on renal manifestations of hospitalized COVID-19–positive patients. However, as other departments raised the need for additional data to address their research questions, the CTRA team proposed an expansion of their data collection efforts which was eventually supported by the Yale DOM. This initiative led to the creation of the DOM-CovX core team which provided access to multi-specialty COVID-19 data as well as guidance on the creation of research protocols and analytic plans [9].

Data on COVID-19–positive hospitalized patients were extracted from the EHR using Clarity (Clarity). The cohort was defined as hospitalizations where the patient’s first positive COVID-19 test was documented 14 days preceding admission to the time of discharge. This definition was chosen to be sufficiently sensitive to capture individuals who may have been tested as an outpatient and then presented for hospitalization 1-2 weeks following diagnosis, while also sufficiently specific to exclude patients with recurrent positive swabs, which may not signify new infection but rather nonviable virus with continued polymerase chain reaction positivity. COVID-19 testing within the YNHHS was done by nasopharyngeal swab testing through polymerase chain reaction.

Data across several key domains were extracted, such as demographics, past medical history, laboratory values during hospitalization, vital signs, medications, imaging, procedures (eg, intubation), and outcomes (eg, death, length of stay, and patient disposition). Given the time-varying nature of several data domains, summary statistics were constructed to limit the computational size of the database and provide a reasonable data file that the broader research community could use for basic statistical analyses. To compress each hospitalization to “one-row-per-patient,” summary statistics were calculated for the time-varying data domains with continuous values, which included vital signs and laboratory values from the respective patient. Summary statistics included minimum, maximum, mean, median, 25th and 75th percentiles, and standard deviation. Medications and procedures were reported as “ever” and “never”; for example, if a patient ever received tocilizumab during hospitalization, the variable would read as a “1” or “0”, respectively.

### Accessing the DOM-CovX Data Explorer and Repository

**Figure 1:**
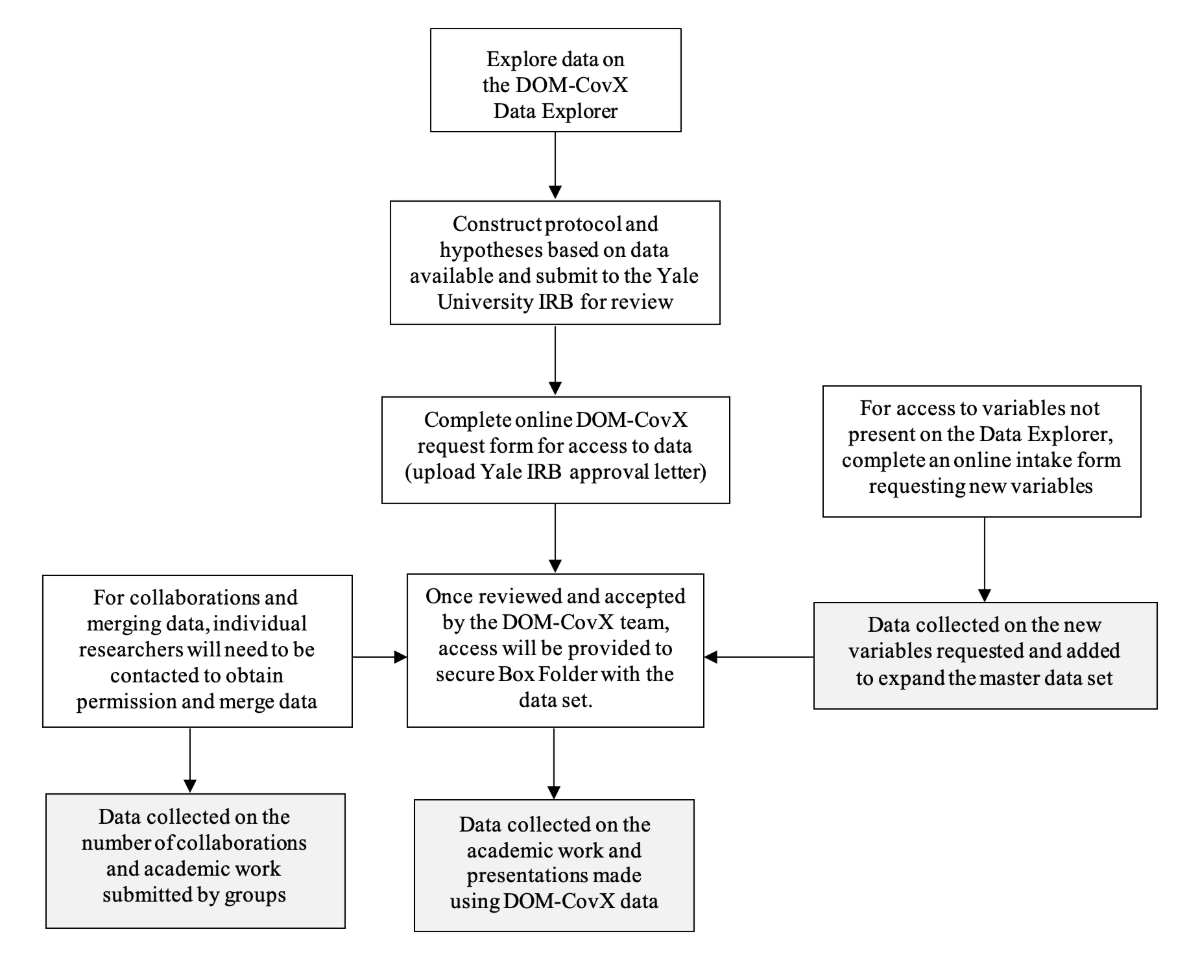
Flowchart of the DOM-CovX data request process. DOM-CovX: Department of Medicine COVID-19 Explorer and Repository; IRB: Institutional Review Board.

The process for accessing the DOM-CovX Data Explorer (web-based platform) and Repository (master data set) is shown in Figure 1. Researchers who wanted to pursue further study had the opportunity to explore a detailed data dictionary to facilitate understanding and transparency of data in the web-based platform and were offered an online form (Multimedia Appendix 1) to request specific data elements from the DOM-CovX Repository as well as information on the population, exposure, outcome of interest, and hypothesis being tested. A core DOM-CovX team consisting of various representatives from the clinical faculty, informatics department, data scientists, statisticians, and research coordinators reviewed the application and assisted in the process. If the team recognized any potential for collaboration between departments and research groups doing similar or related projects, a dialogue would be facilitated.

Researchers interested in using the DOM-CovX Repository used this algorithm to gain access. Key steps include initial submission to the Yale University institutional review board (IRB), completion of the online DOM-CovX request form, and review of the application by the DOM-CovX team. Requests for new data variables and collaborations were also part of the review. Once the application was accepted, access was granted to a Secure Box folder, a HIPAA (Health Insurance Portability and Accountability Act)-compliant cloud-based file management system used at Yale for data distribution.

### Data Distribution

Once the application was approved by the core DOM-CovX team, data were distributed to researchers through Secure Box. Data were left in raw format and used by researchers on a variety of statistical software packages for analysis. Biostatistical analysis was left to the individual researchers but in case they would need any assistance with the process, help would be provided by the DOM-CovX team. The analysis process could include merging of DOM-CovX data to another data collection and analytical systems, such as REDCap (Research Electronic Data Capture; Vanderbilt University), a browser-based metadata-driven software to collect and store data for clinical and translational research [10,11]. Researchers had the option of requesting additional data elements from the DOM-CovX team as well as engaging in manual data extraction for fields that were not amenable to computerized data transfer; these additional data elements were also added to the master data set. A master file for all requests and data provisions was maintained and monitored to determine any potential for facilitating collaborations. The DOM-CovX team also tracked resulting presentations, publications, and other academic productivity.

### Front-Facing Web Interface for Data Exploration (DOM-CovX Explorer)

**Figure 2:**
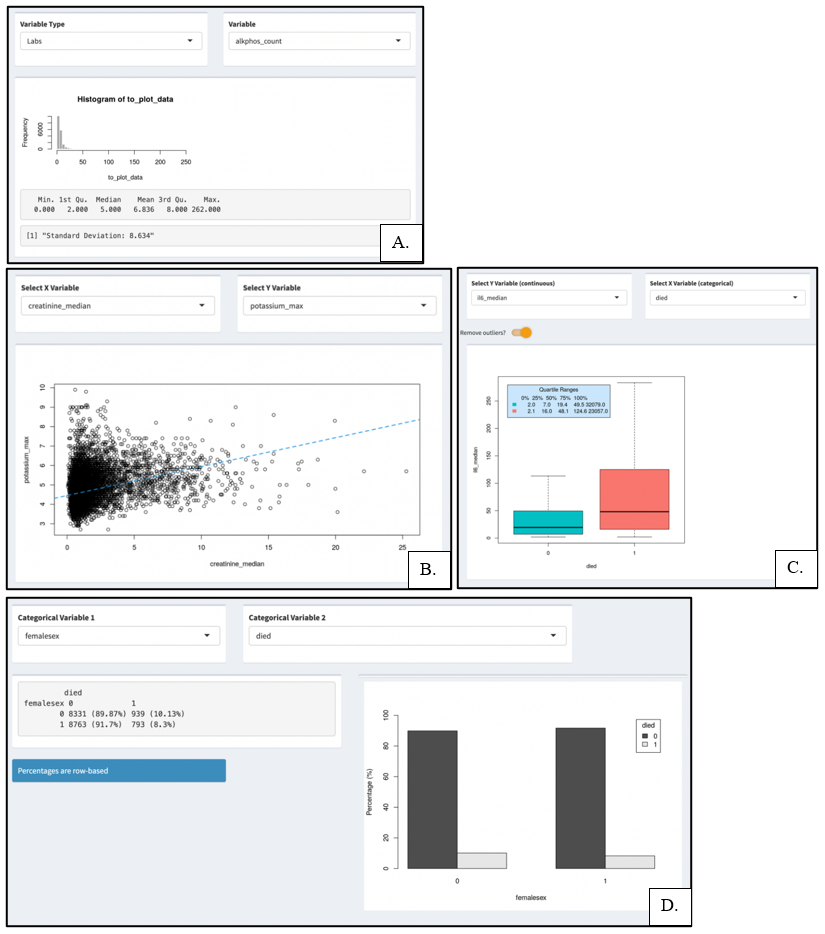
Web interface of the DOM-CovX Explorer—sample queries extracted in October 2022, showing different functions of Explorer to find correlations between variables and aid in hypothesis generation. (A) Histogram showing frequency of patients with variable “alkaline phosphatase” available in the data set. (B) Scatter plot showing correlation between the independent variable “median creatinine” (plotted in x-axis) and the dependent variable “maximum potassium” (plotted in y-axis). (C) Box Plot displaying the distribution of the variable “median IL-6” in patients who died (1) and did not die (0). (D) 2x2 Table and bar graph showing the frequency and proportion of patients by sex (female=1, male=0) that died (1) and did not die (0). DOM-CovX: Department of Medicine COVID-19 Explorer and Repository.

A unique feature of this initiative was the DOM-CovX Explorer, a web-based app designed to provide investigators with a front-end interface to view and inspect variables, see summary statistics, and receive additional information about cohort design and data requests. It was constructed using R Shiny (R Foundation for Statistical Computing) and the statistical visualizations included single variable analyses, scatter plots, box plots, as well as 2×2 tables as shown in Figure 2*.* Yale researchers have been able to openly explore the data to assess if they are interested in further in-depth study that requires access to the data repository.

### Ethical Considerations

A preapproved Human Investigations Committee number and a Yale IRB protocol were required to be uploaded at the time of data request. In case researchers did not have approval yet, the explorer offered a downloadable Yale IRB template for guidance and rapid institutional approval of research protocols. The Yale IRB reviews proposed studies regarding the need for informed consent and this is detailed in the separate process for access to DOM-CovX. Specifically, for secondary data analyses, the IRB grants waivers in line with specific criteria. The IRB application process also details candidates’ compensation when relevant.

For the repository, data were deidentified to remove protected health information as per the HIPAA Security Rule. All dates more specific than year were removed including admission and discharge dates and times, timing of vital signs, laboratory measurements, procedures, or medication administrations. Ages greater than 90 years were set to 90 as per the HIPAA Security Rule. This initiative was reviewed and approved by the Yale IRB (Protocol ID 2000028509)

## Results

### Uptake of the DOM-CovX Database Over Time

Since its creation, the DOM-CovX team has received 38 requests from different groups of scientists at Yale to access the database with their research and clinical teams. Most of the requests occurred during the first wave of the pandemic; however, the team has continued receiving at least 1 request every 2-3 months until the present.

Since its creation, the database has included more than 3000 clinically relevant data elements such as demographics, laboratory values, past medical history, vital signs, and medications. The database covers the vast majority of EHR data variables, including specifics of disease stages (eg, acute kidney injury stage 1, 2, and 3), distribution of data elements (eg, mean, mode, median, SD, and IQRs), as well as the addition of data elements generated by automated data extraction or chart review as requested by researchers. The database is regularly updated in a monthly fashion and since July 2020, it has expanded to include 18,828 inpatient encounters, with up to 3997 variables across 7 different clinical domains, which includes added variables related to the hospitalization (eg, length of stay and type of hospital unit).

### Use of the DOM-CovX by Specialty

The DOM-CovX Data Explorer and Repository have been easily accessed and completely free of charge to all Yale faculty and trainees who may otherwise lack independent grant funding and staff to pursue data repository creation. The DOM-CovX has been used by 15 different specialties within the Internal Medicine department, as well as other departments such as Anesthesiology, Neurology, Obstetrics and Gynecology, Pathology, Radiology, and Surgery. Out of the total data requests that we have received, 79% (30/38) have been from different nonsurgical specialties and 21% (8/38) from different surgical specialties (Multimedia Appendix 2). The database has been used to address diverse questions, such as (1) the impact of obesity and diabetes [12], (2) the association of acute kidney injury with COVID-19 [13], (3) the role of neutrophil activation signature in predicting critical illness and mortality in COVID-19 [14], and (4) the association of HIV with COVID-19 outcomes [15].

The simplicity of data request and acquisition, and encouragement to collaborate with established faculty have allowed early career trainees to formulate hypotheses, receive timely access to the repository, and receive mentorship in different areas of interest (Multimedia Appendix 3). The range of users of the database has included 57% (39/68) individuals in a faculty position, 19% (13/68) clinical trainees, 7% (5/68) students, and 16% (11/68) individuals in other research positions.

### Academic Productivity Derived From the DOM-CovX Database

Multiple collaborations between different departments have been made, bringing together researchers from Cardiology, Emergency Medicine, Endocrinology, Hematology, and Oncology. From January 2020 to January 2023, 18 papers (16 peer-reviewed and 2 in preprints) were published using DOM-CovX data, and 8 abstracts were accepted for presentation in different scientific conferences (Multimedia Appendix 4).

## Discussion

### Principal Findings

The Yale DOM-CovX, an innovative platform with data from hospitalized patients with COVID-19 in the YNHHS, has been extensively used for a wide range of scholarly productivity during the recent pandemic. It has promoted interdepartmental collaborations and encouraged early career trainees to receive mentorship through a comprehensive, regularly updated, and easily accessed data set. Data requests have involved 38 different groups of scientists and trainees and have resulted in over 20 publications and scientific conference presentations.

During the COVID-19 pandemic, there has been an increase in the availability of open-access data. Clinicians and researchers have been able to access online data trackers from Johns Hopkins, the CDC COVID data tracker, ClinicalTrials.gov website, and local health departments [16-18]. Some of these databases offer summarized information on case trends, demographics, vaccinations, and other outcomes; however, specific clinically relevant and individual patient-level data are often lacking [19,20]. Larger national and international databases with clinical data such as the National COVID Cohort Collaborative (N3C) created and maintained by the National Institutes of Health (NIH), the Infectious Diseases Data Observatory (IDDO), and the International Severe Acute Respiratory and Emerging Infection Consortium (ISARIC) are available, but access can be restricted [21-24]. Local proprietary databases, such as the COVID-19–specific Johns Hopkins Crown Registry (JH-CROWN), use a precision medicine analytics platform that aims to distribute data to institutional investigators [25-27]. Researchers have also created large-scale data repositories for COVID-19 including an alternative COVID-19 clinical data repository curated through the Observational Medical Outcomes Partnership (OMOP) initiative [28,29]. However, these repositories are primarily accessible to large research teams that have the expertise of exploring and analyzing intricated data sets and can present logistic barriers for other faculty and trainees who do not have access to it. In contrast, one significant advantage of the comprehensive DOM-CovX is the availability of detailed clinical variables from hospitalized patients with COVID-19 within our geographic location. Locally relevant research that has significant community impact can be made accessible.

Facilitating the research process is another major benefit of the DOM-CovX Explorer. This website allows the inspection of variables, an easy application process to access as well as support in project design and data analysis if needed. This leads us to one of the lessons learned from the development of this initiative. The use of internal resources facilitates prompt access to data and gives us the flexibility to generate timely changes to the data set if needed. We encourage institutions and their researchers to invest in internal resources considering how responsive these are to local needs and further input. Currently, access is offered to all Yale clinicians and researchers and potential expansion of its availability to researchers outside the institution is being considered. This new step could lead the way to institutional collaborations within and outside the United States to continue evaluating the epidemiology of the COVID-19 pandemic.

We anticipate that as the process evolves, there will be a growing impact on medical education and scientific productivity involving researchers in different stages of training like we have evidenced locally. Specifically, the rapidly changing academic landscape requires that curricula provide the intellectual currency for all trainees and faculty to critically understand the explosion of clinical and research literature including the optimal use of data and recognition of its limitations. Inequities of data access may result in a more divided group of learners with narrower expectations and rewards. DOM-CovX presents a basis for further generation of databases or repositories focused on bridging the intellectual divide and expanding the scope and quality of medical education. Thus, another lesson learned is the importance of establishing strategies to promote access to the resources that expand knowledge and promote education. For DOM-CovX, these strategies are an ongoing effort that aims to secure the sustainability of this initiative and promote its constant growth.

Currently, tools for statistical analysis have not yet been built into the workflow. Nonetheless, future goals could incorporate access to fundamental analytic tools and statisticians, improve training and mentoring of clinician-educators, and aid clinicians to engage in fundamental understanding of research analytics as well as to improve data management skills. The open access of DOM-CovX could also improve the implementation of available analytic resources. Zanettini et al [30] presented one possibility with the COVID-19 census, an R package that retrieves different repositories and databases from the United States and Italy, creating a combined set of metrics and other demographic variables, to facilitate multivariable analysis and modeling by the scientific community.

Limitations of the DOM-CovX Repository are inclusion only of data on hospitalized patients. The database focuses only on clinical data and does not lend itself to studies requiring a biorepository.

### Conclusion

The Yale DOM-CovX Data Explorer and repository have provided an accessible tool for simple data analysis and access to a consistently updated, standardized, and large-scale database for patients hospitalized with COVID-19 in our hospital system. It overcomes barriers for a wide variety of researchers and has demonstrated the potential to increase academic productivity. It represents an opportunity to improve the institutional climate by fostering collaboration and diversity of scholarly pursuits.

## Appendix

Multimedia Appendix 1 DOM-CovX data request form for researchers affiliated at Yale University. DOM-CovX: Department of Medicine COVID-19 Explorer and Repository.

Multimedia Appendix 2 Department of Medicine COVID-19 Explorer and Repository requests by specialty at Yale School of Medicine from January 2020 to January 2023.

Multimedia Appendix 3 Academic positions of researchers at Yale School of Medicine that requested access to the Department of Medicine COVID-19 Explorer and Repository from January 2020 to January 2023.

Multimedia Appendix 4 Academic progress from Yale School of Medicine departments using DOM-CovX data repository from 1/2020-1/2023. DOM-CovX: Department of Medicine COVID-19 Explorer and Repository.

## Abbreviations

CTRA: clinical and translational research accelerator
DOM: Department of Medicine
DOM-CovX: Department of Medicine COVID-19 Explorer and Repository
EHR: electronic health record
HIPAA: Health Insurance Portability and Accountability Act
IDDO: Infectious Diseases Data Observatory
IRB: Institutional Review Board
ISARIC: International Severe Acute Respiratory and Emerging Infection Consortium
JH-CROWN: Johns Hopkins Crown Registry
N3C: National COVID Cohort Collaborative
NIH: National Institutes of Health
OMOP: Observational Medical Outcomes Partnership
REDCap: Research Electronic Data Capture
YNHHS: Yale New Haven health system
YSM: Yale University School of Medicine

## Data Availability

Data sharing is currently not applicable as access to the Department of Medicine COVID-19 Explorer and Repository is restricted to the YSM community. However, individual researchers who publish their studies may grant access to their databases upon special request.
