# Supplementary material for: Promoting Collaborative Scholarship During the COVID-19 Pandemic Through an Innovative COVID-19 Data Explorer and Repository at Yale School of Medicine: Development and Usability Study
Source: JMIR Form Res. 2024 Sep 3;8:e52120. doi: 10.2196/52120 (PMC11408881; doi:10.2196/52120)
Supplement: Multimedia Appendix 1 [file formative_v8i1e52120_app1.docx]

**DOM-COVX Data Request Form**

**Q1.** Title of Research Proposal

**Q2.** First name of Principle Investigator

**Q3.** Last name of Principle Investigator

**Q4.** NetID of Principle Investigator

**Q5.** Email of Principle Investigator

**Q6.** Would you like to add any Co-Investigators?

**Q7.** Population(s) of Interest

**Q8.** Exposure(s) of Interest

**Q9.** Outcome(s) of Interest

**Q10.** Hypothesis and/or Statement of Intent

**Q11.** Approved project Human Investigation Committee (HIC) #

**Q12.** Please upload your Institutional Review Board (IRB)-approved protocol

**Q13.** Will your team require biostatistics support?

**Q14.** Will your team require data including personal health information (PHI)? (e.g. patient identifiers such as medical record numbers (MRNs), zip codes etc.)

**Q15.** From the following sub-categories, please select ALL relevant to your data needs.

**Q16.** Will the data you are requesting be combined with novel data you or your collaborators have generated as part of a research project?

**Q17.** Will data you receive by DOM-CovX team be sent, used, or analyzed by any non-Yale institutions, companies, contractors or non-Yale affiliated individuals?

**Q18.** Would you like to provide any additional notes/comments?
