# Supplementary figures and images for: Promoting Collaborative Scholarship During the COVID-19 Pandemic Through an Innovative COVID-19 Data Explorer and Repository at Yale School of Medicine: Development and Usability Study
Source: JMIR Form Res. 2024 Sep 3;8:e52120. doi: 10.2196/52120 (PMC11408881; doi:10.2196/52120)

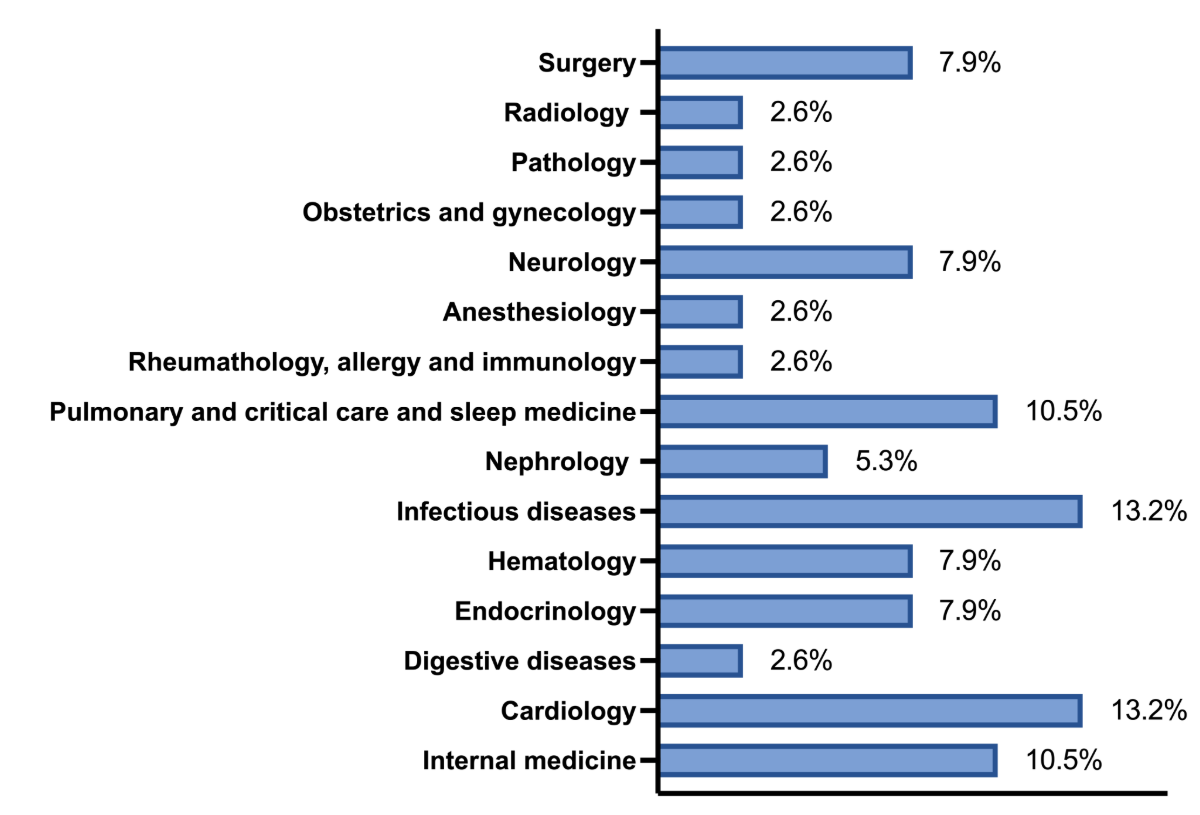

Supplement: Multimedia Appendix 2 [file formative_v8i1e52120_app2.png]

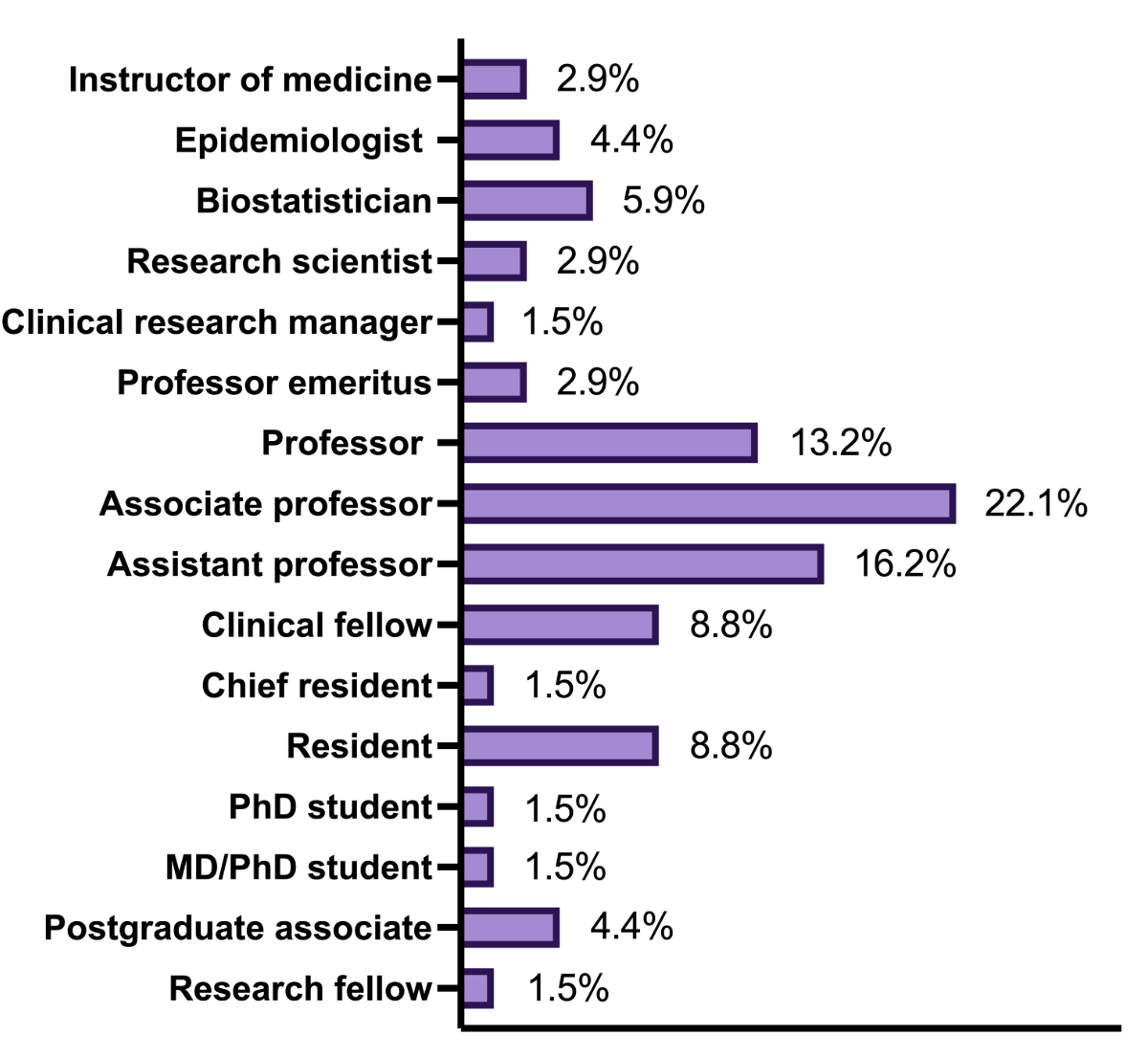

Supplement: Multimedia Appendix 3 [file formative_v8i1e52120_app3.png]
