# Supplementary material for: Promoting Collaborative Scholarship During the COVID-19 Pandemic Through an Innovative COVID-19 Data Explorer and Repository at Yale School of Medicine: Development and Usability Study
Source: JMIR Form Res. 2024 Sep 3;8:e52120. doi: 10.2196/52120 (PMC11408881; doi:10.2196/52120)
Supplement: Multimedia Appendix 4 [file formative_v8i1e52120_app4.docx]

| **Department** | **Authors** | **Title of project** | **Type of publication** | **Journal and year of publication** |
| --- | --- | --- | --- | --- |
| **Cardiology** | Kwan JM, et al. [31] | Cardiovascular and mortality outcomes of hospitalized COVID-19 patients stratified by race and ethnicity | Accepted abstract | Journal of the American College of Cardiology. 2021 |
|  | Kwan JM, et al. [32] | Cardiovascular and mortality outcomes in oncology patients hospitalized with COVID-19 | Accepted abstract | Journal of the American College of Cardiology. 2021 |
| **Emergency Medicine** | Haimovich AD, et al. [33] | Development and validation of the quick covid-19 severity index: a prognostic tool for early clinical decompensation | Peer reviewed – Original article | Annals of Emergency Medicine. 2020 |
|  | Haimovich AD, et al. [34] | Patient factors associated with SARS-CoV-2 in an admitted emergency department population | Peer reviewed – Brief research report | Journal of the American College of Emergency Physicians Open. 2020 |
| **Endocrinology** | Jastreboff A. et al. [12] | Impact of Obesity and Diabetes on COVID-19 outcomes | Oral abstract presentation | Endocrine Society Conference. 2020 |
|  | Kang YM, et al. [35] | Obesity is associated with intensive care use and duration of ICU stay but not mortality among 3246 patients hospitalized with COVID-19 | Accepted abstract | Journal of the Endocrine Society. 2021 |
|  | Brancale J, et al. [36] | The impact of baseline A1C and admission glucose on mortality among patients with diabetes hospitalized with COVID-19 | Accepted abstract | Diabetes. 2021 |
| **Hematology** |  |  |  |  |
|  | Meizlish ML, et al. [14] | A neutrophil activation signature predicts critical illness and mortality in COVID-19 | Peer reviewed – Original article | Blood Advances. 2021 |
|  | Wang SY, et al. [37] | Challenges in interpreting cytokine data in COVID-19 affect patient care and management | Peer reviewed – Perspective article | PLOS Biology. 2021 |
| **Hepatology** | McConnell MJ, et al. [38] | Liver injury in COVID-19 and IL-6 trans-signaling-induced endotheliopathy | Peer reviewed – Original article | Journal of Hepathology. 2021 |
| **Infectious diseases** | Virata M, et al. [15] | Outcomes Related to COVID-19 Among People Living with HIV: Cohort from a Large Academic Center | Accepted abstract | Open Forum Infectious Diseases. 2020 |
| **Nephrology** | Moledina DG and Simonov M, et al. [13] | The association of COVID-19 with acute kidney injury independent of severity illness: a multicenter cohort study | Peer reviewed – Original article | American Journal of Kidney Diseases. 2021 |
|  | Mansour S, et al. [39] | A comparison study of COVID-19 outcomes in hospitalized kidney transplant recipients | Peer reviewed – Original article | Kidney360. 2021 |
|  | Nugent J, et al. [40] | Assessment of acute kidney injury and longitudinal kidney function after hospital discharge among patients with and without COVID-19 | Peer reviewed – Original article | JAMA Network Open. 2021 |
|  | Ghazi L, et al. [41] | Predicting patients with false negative SARS-CoV-2 testing at hospital admission: a retrospective multi-center study | Peer reviewed – Original article | PLOS ONE. 2021 |
| **Vascular Medicine** | Smolderen KG, et al [42] | Peripheral artery disease and COVID-19 outcomes: insights from the Yale DOM-CovX Registry | Peer reviewed – Original article | Current Problems in Cardiology. 2021 |
|  | Smolderen KG, et al. [43] | Peripheral artery disease and SARS-CoV-2 outcomes in the Yale DOM-CovX Registry | Accepted abstract | Journal of the American College of Cardiology. 2021 |
| **Surgery** | Kodadek LM, et al [44] | Palliative Care in a Pandemic: A Multicenter Cohort of Critically Ill Patients with Coronavirus Disease 2019 | Peer reviewed – Original article | Surgical Infections. 2023 |
| **Multi-collaboration** | Datta R, et al. [45] | Active monitoring of a healthcare worker cohort during the COVID-19 epidemic | Accepted abstract | Open Forum Infectious Diseases. 2020 |
|  | Kuchroo M, et al. [46] | Multiscale PHATE exploration of SARS-CoV-2 data reveals multimodal signatures of disease | Preprint | bioRxiv. 2020 |
|  | Shin JJ, et al. [47] | A distinct association of inflammatory molecules with outcomes of COVID-19 in younger versus older adults | Peer reviewed – Original article | Clinical Immunology. 2021 |
|  | Amodio M, et al. [48] | Generating hard to obtain information from easy to obtain information: applications in drug discovery and clinical inference | Peer reviewed – Original article | Patterns. 2021 |
|  | Kuster JK, et al. [49] | Low IgG trough and lymphocyte subset counts are associated with hospitalization for COVID-19 in patients with primary antibody deficiency | Peer reviewed – Clinical communication | Journal of Allergy and Clinical Immunology. 2022 |
|  | Safdar B, et al. [50] | Association of renalase with clinical outcomes in hospitalized patients with COVID-19 | Peer reviewed – Original article | PLOS ONE. 2022 |
|  | Kuchroo M, et al. [51] | Multiscale PHATE identifies multimodal signatures of COVID-19 | Peer reviewed – Original article | Nature Biotechnology. 2022 |

**REFERENCES**

31. Kwan J, Lee S, Tao W, et al. CARDIOVASCULAR AND MORTALITY OUTCOMES OF HOSPITALIZED COVID-19 PATIENTS STRATIFIED BY RACE AND ETHNICITY. *Journal of the American College of Cardiology*. 2021;77(18):3050. doi:10.1016/S0735-1097(21)04405-3

32. Kwan J, Lee S, Tao W, et al. CARDIOVASCULAR AND MORTALITY OUTCOMES IN ONCOLOGY PATIENTS HOSPITALIZED WITH COVID-19. *Journal of the American College of Cardiology*. 2021;77(18):3294. doi:10.1016/S0735-1097(21)04648-9

33. Haimovich AD, Ravindra NG, Stoytchev S, et al. Development and Validation of the Quick COVID-19 Severity Index: A Prognostic Tool for Early Clinical Decompensation. *Annals of Emergency Medicine*. 2020;76(4):442-453. doi:10.1016/j.annemergmed.2020.07.022

34. Haimovich AD, Warner F, Young HP, et al. Patient factors associated with SARS‐CoV‐2 in an admitted emergency department population. *Journal of the American College of Emergency Physicians Open*. 2020;1(4):569-577. doi:10.1002/emp2.12145

35. Kang YM, Brancale J, Athonvarangkul D, et al. Obesity Is Associated With Intensive Care Use and Duration of ICU Stay but Not Mortality Among 3246 Patients Hospitalized With COVID-19. *Journal of the Endocrine Society*. 2021;5(Supplement_1):A61-A62. doi:10.1210/jendso/bvab048.125

36. Brancale J, Athonvarangkul D, Simonov M, et al. 1014-P: The Impact of Baseline A1C and Admission Glucose on Mortality among Patients with Diabetes Hospitalized with COVID-19. *Diabetes*. 2021;70(Supplement_1):1014-P. doi:10.2337/db21-1014-P

37. Wang SY, Takahashi T, Pine AB, et al. Challenges in interpreting cytokine data in COVID-19 affect patient care and management. *PLoS Biol*. 2021;19(8):e3001373. doi:10.1371/journal.pbio.3001373

38. McConnell MJ, Kawaguchi N, Kondo R, et al. Liver injury in COVID-19 and IL-6 trans-signaling-induced endotheliopathy. *Journal of Hepatology*. 2021;75(3):647-658. doi:10.1016/j.jhep.2021.04.050

39. Mansour SG, Malhotra D, Simonov M, et al. A Comparison Study of Coronavirus Disease 2019 Outcomes in Hospitalized Kidney Transplant Recipients. *Kidney360*. 2021;2(3):494-506. doi:10.34067/KID.0005652020

40. Nugent J, Aklilu A, Yamamoto Y, et al. Assessment of Acute Kidney Injury and Longitudinal Kidney Function After Hospital Discharge Among Patients With and Without COVID-19. *JAMA Netw Open*. 2021;4(3):e211095. doi:10.1001/jamanetworkopen.2021.1095

41. Ghazi L, Simonov M, Mansour SG, et al. Predicting patients with false negative SARS-CoV-2 testing at hospital admission: A retrospective multi-center study. Lazzeri C, ed. *PLoS ONE*. 2021;16(5):e0251376. doi:10.1371/journal.pone.0251376

42. Smolderen KG, Lee M, Arora T, Simonov M, Mena-Hurtado C. Peripheral Artery Disease and COVID-19 Outcomes: Insights from the Yale DOM-CovX Registry. *Current Problems in Cardiology*. Published online October 2021:101007. doi:10.1016/j.cpcardiol.2021.101007

43. Smolderen KG, Nagpal S, Simonov M, Arora T, Hurtado CM. PERIPHERAL ARTERY DISEASE AND SARS-COV-2 OUTCOMES IN THE YALE DOM-COVX REGISTRY. *Journal of the American College of Cardiology*. 2021;77(18):1834. doi:10.1016/S0735-1097(21)03190-9

44. Kodadek LM, Moore MS, Miller SM, et al. Palliative Care in a Pandemic: A Multicenter Cohort of Critically Ill Patients with Coronavirus Disease 2019. *Surgical Infections*. Published online February 8, 2023:sur.2022.377. doi:10.1089/sur.2022.377

45. Datta R, Campbell M, Wyllie A, et al. 68. Active Monitoring of a Healthcare Worker Cohort During the COVID-19 Epidemic. *Open Forum Infectious Diseases*. 2020;7(Supplement_1):S165-S165. doi:10.1093/ofid/ofaa439.378

46. Kuchroo M, Huang J, Wong P, et al. *Multiscale PHATE Exploration of SARS-CoV-2 Data Reveals Multimodal Signatures of Disease*. Bioinformatics; 2020. doi:10.1101/2020.11.15.383661

47. Shin JJ, Jeon S, Unlu S, et al. A distinct association of inflammatory molecules with outcomes of COVID-19 in younger versus older adults. *Clinical Immunology*. 2021;232:108857. doi:10.1016/j.clim.2021.108857

48. Amodio M, Shung D, Burkhardt DB, et al. Generating hard-to-obtain information from easy-to-obtain information: Applications in drug discovery and clinical inference. *Patterns*. 2021;2(7):100288. doi:10.1016/j.patter.2021.100288

49. Kuster JK, Unlu S, Makin TA, et al. Low IgG trough and lymphocyte subset counts are associated with hospitalization for COVID-19 in patients with primary antibody deficiency. *The Journal of Allergy and Clinical Immunology: In Practice*. 2022;10(2):633-636.e3. doi:10.1016/j.jaip.2021.11.030

50. Safdar B, Wang M, Guo X, et al. Association of renalase with clinical outcomes in hospitalized patients with COVID-19. Torti C, ed. *PLoS ONE*. 2022;17(3):e0264178. doi:10.1371/journal.pone.0264178

51. Kuchroo M, Huang J, Wong P, et al. Multiscale PHATE identifies multimodal signatures of COVID-19. *Nat Biotechnol*. 2022;40(5):681-691. doi:10.1038/s41587-021-01186-x
